# Phenotypic, biochemical and genomic variability in generations of the rapeseed (*Brassica napus* L.) mutant lines obtained via chemical mutagenesis

**DOI:** 10.1371/journal.pone.0221699

**Published:** 2019-08-28

**Authors:** Alexandra V. Amosova, Svyatoslav A. Zoshchuk, Valentina T. Volovik, Anna V. Shirokova, Nickolai E. Horuzhiy, Galina V. Mozgova, Olga Yu. Yurkevich, Margarita A. Artyukhova, Valentina A. Lemesh, Tatiana E. Samatadze, Olga V. Muravenko

**Affiliations:** 1 Engelhardt Institute of Molecular Biology, Russian Academy of Sciences, Moscow, Russian Federation; 2 Federal Williams Research Center of Forage Production and Agroecology, Lobnya, Moscow region, Russian Federation; 3 Koltzov Institute of Developmental Biology, Russian Academy of Sciences, Moscow, Russian Federation; 4 Institute of Genetics and Cytology, National Academy of Sciences of Belarus, Minsk, Belarus; Agriculture and Agri-Food Canada, CANADA

## Abstract

The phenotypic, biochemical and genetic variability was studied in M2-M5 generations of ethyl methansulfonat (EMS, 0.2%) mutagenized rapeseed lines generated from canola, ‘00’, *B*. *napus* cv. Vikros. EMS mutagenesis induced extensive diversity in morphological and agronomic traits among mutant progeny resulted in selection of EMS populations of *B*. *napus*- and *B*. *rapa-*morphotypes. The seeds of the obtained mutant lines were high-protein, low in oil and stabilized in contents of main fatty acids which make them useful for feed production. Despite the increased level of various meiotic abnormalities revealed in EMS populations, comparative karyotype analysis and FISH-based visualization of 45S and 5S rDNA indicated a high level of karyotypic stability in M2-M5 plants, and therefore, the obtained mutant lines could be useful in further rapeseed improvement. The revealed structural chromosomal reorganizations in karyotypes of several plants of *B*. *rapa-*type indicate that rapeseed breeding by chemical mutagenesis can result in cytogenetic instability in the mutant progeny, and therefore, it should include the karyotype examination. Our findings demonstrate that EMS at low concentrations has great potential in rapeseed improvement.

## Introduction

Rapeseed (*Brassica napus* L.) is one of the most economically important crops widely used in different industries as an important source of edible vegetable oil, animal fodder and biodiesel [1, 2]. *B*. *napus* is considered to be a natural amphidiploid (genome AACC, 2n = 38) originated from spontaneous hybridization between the ancestors of *B*. *rapa* L. (AA; 2n = 20) and *B*. *oleracea* L. (CC; 2n = 18) followed by diploidization [3–5]. The polyphyletic origin of *B*. *napus* has also been confirmed by results of organelle and nuclear RFLP analyses [6]. Although both *B*. *oleracea* and *B*. *rapa* have a great diversity of morphotypes with various origins, *B*. *napus* is characterized by a relatively narrow genetic diversity [7, 8]. Moreover, breeding selection resulted in a decrease of genetic basis of current rapeseed cultivars. Therefore, new genetic sources and approaches are needed to diversify the genetic basis of rapeseed germplasm, which will make the current breeding programs more effective [9, 10]. Examples of such approaches may include intraspecific hybridization and a recombinant DNA technology [11, 12], creation of synthetic rapeseed lines via artificial crosses between various *Brassica* species containing A and C genomes [13–15] and also chemical and physical mutagenesis [16–17]. Chemical mutagenesis is an effective and simple method for obtaining valuable starting material that can further be used in crop improvement programs [17, 18]. Chemical mutagens (e.g., azide, diethyl sulphate, dimethyl sulphate, ethyl methanesulphonate and N-nitroso compounds) are known to induce non-lethal point DNA mutations at a high rate and create novel genetic diversity in various crops [16, 19–22]. Particularly, this approach is widely used in rapeseed breeding to produce new cultivars with the desired morpho-agronomic traits and/or biochemical profile which are difficult to obtain though crossbreeding and selection [11, 12, 23].

The fatty acid biosynthesis pathway is a primary metabolic pathway in oil-bearing plants [24]. Acetyl-CoA is the basic component of the fatty acid chain, involved in the synthesis of 16- or 18-carbon products, which are the major (up to 90%) fatty acids in plants. Various desaturases located in the plastids and the endoplasmic reticulum are responsible for catalyzing these fatty acids to become monounsaturated (palmitoleic acid, C_16:1_, and C_18:1_) or polyunsaturated ones (C_18:2_ and C_18:3_). The fatty acid composition of the rapeseed oil is the main trait determined its utilization mode and range [25]. Seeds of the double-low varieties (canola, ‘00’, with very low glucosinolates and erucic acid content) produce oil containing approximately 7% of saturated fatty acids (including palmitic (C_16:0_) and stearic (C_18:0_)), 61% of the monounsaturated oleic acid (C_18:1_) and polyunsaturated fatty acids (linoleic (C_18:2_, 20%), linolenic (C_18:3_, 10%) and eicosenoic (C_20:1_, 1%)). This fatty acid composition is considered optimal for nutritional purposes [26]. However, due to the food- and non-food use of the oil, the demand for rapeseed oils with other fatty acid compositions exists in the market [11, 27–29].

The investigation of mutant rapeseed genomes is mostly related to the allele polymorphism analysis and mapping of the mutant genes associated to agronomic traits. The content of erucic acid in *B*. *napus* is found to be under additive control of alleles of FAE1.1 and FAE1.2 genes encoding the enzyme of erucic acid synthesis, 3-ketoacyl-CoA synthase, from the oleoyl-CoA [30, 31]. It was shown that loss of functions of FAE1.2 (C subgenome) and one base pair substitution in FAE1.1 gene (A subgenome) led to formation of canola, ‘00’, plants [32, 33]. The content of oleic acid is controlled by the fatty acid desaturase 2 (FAD2) gene that encodes endoplasmic delta-12 fatty acid desaturase 2 (112-FAD2) which converts the precursors of oleic acid to the precursors of linoleic acid in the lipid biosynthetic pathway [24, 34–36]. Four homologous FAD2 genes (BnFAD2-1, BnFAD2-2, BnFAD2-3, and BnFAD2-4) located separately on rapeseed chromosomes of A and C subgenomes, and their possible role in the rapeseed genome is oleic acid regulation [37, 38]. The linolenic acid content in *B*. *napus* is controlled by two fatty acid desaturase 3 (FAD3) genes (BnaA.FAD3 and BnaC.FAD3), encoding delta-15 linoleate desaturase which is responsible for dehydration of linoleic acid to linolenic acid [39]. These genes were detected in the A and C subgenomes of *B*. *napus* [40] and mapped in the N4 (A4) and N14 (C4) linkage groups, correspondingly [39].

More variability of rapeseed germplasms can be created via mutagenesis [23, 36, 39, 41–42]. At the same time, experimental mutagenesis in allopolyploid *B*. *napus* might result in various genetic, chromosomal and genomic reorganizations promoting genetic instability in the progeny. However, in karyotypes of rapeseed mutants, the structure of chromosomes and possible intra- and intergenomic structural rearrangements and substitutions are poorly investigated. Due to small rapeseed chromosomes (1.53–3.30 μm) [43], the detailed chromosomal analysis is still problematic and needs special approaches, e.g., chromosome elongation with the use of DNA intercalators, application of chromosomal markers allowing identification of individual rapeseed chromosomes and their subgenomic affiliation [44–47]. Comprehensive study of the genotypic variability in mutant rapeseed lines in combination with the karyotype structure analysis (chromosomal complements in A and C subgenomes, the presence of chromosome rearrangements, chromosome substitutions and additions), description of phenotypic and biochemical variability was not performed. Integration of mutation techniques with the molecular, cytogenetic and biochemical analyses provides exciting opportunities for rapeseed breeding. Such approach could be useful in developing reliable tools for improving selection methods and also for introducing novel traits into rapeseed cultivars.

The objectives of the present study were to analyze phenotypic, biochemical and cytogenomic variability in M1-M5 generations of the ethyl methanesulfonate (EMS) mutagenized progeny of the spring canola *B*. *napus* cv. Vikros in order to reveal agronomically valuable and genetically stable rapeseed mutant genotypes. The current approach based on the analysis of morphological and agronomic traits, the biochemical profile, SNaPshot detection of mutant and wild-type FAD3 genes, meiosis and FISH localization of 5S and 5S rDNA has been applied.

## Materials and methods

### Ethics statement

This study including plant sample collection and experimental research conducted on these materials was according to the federal law on environmental protection approved by the Council of the Russian Federation.

### Plant material

Seeds of the spring canola, ‘00’, *B*. *napus* cv. Vikros (3480, Russian Federation) were obtained from the germplasm collections of Federal Williams Research Center of Forage Production and Agroecology, Lobnya, Moscow, Russian Federation. Before the mutagenesis assays, the progeny of three succeeding generations (I1-I3) of this *B*. *napus* cv. Vikros was tested for hidden effects of inbreeding (self-pollination), and no deviations from the standard characteristics of the original cultivar were revealed. To diversify the genetic basis of the rapeseed germplasm, the seeds of the original cultivar were treated with aqueous solution of ethyl methanesulphonate in concentrations of 0.2% for 16 h. All studied rapeseed plants were grown with the use of pre-grown seedlings: seeds were sowing in the greenhouse followed (30–40 days later) the outdoor planting of at least 50 seedlings. In mutant plants, the leading shoots were isolated at floral initiation stage to obtain self-pollinated seeds. At maturity, seed siliqua were collected from the leading shoots. The identification of the plants was performed according to the Manual of *Brassica napus* L. [48]. The progeny selection for morphological and agronomic characters was carried out in М2-М5 plants. Statistical data analysis was performed using standard functions of Microsoft Excel 2013. For each generation, at least 50 plants in every mutant line were analyzed.

### Biochemical profile

The biochemical profile was analysed for 20 plants of each mutant line. The fatty acid composition and total oil content were determined in milled seeds (2 g from one plant, 15 plants of each line) using the gas chromatograph Kristall 2000M (Chromateck, Yoshkar Ola, RF) with Zebron ZB-FFAP Capillary GC Column 25m x 0.20mm x 0.30μm (Phenomenex, Torrance, USA) according to the manufacturer’s protocol. The content of protein was estimated photometrically using a biochemical flowing auto analyzer of chemical composition CIAK-K (Kinzh-Agro, Moscow, RF) according to the manufacturer’s protocol. Statistical data analysis was performed using standard functions of Microsoft Excel 2013.

### DNA extraction

Total genomic DNA was extracted from green leaves and seedlings using the “Genomic DNA Purification Kit” (Thermo Fisher Scientific, Vilnius, Lithuania) according to the manufacturer’s protocol. The DNA concentration and purification degree were determined using the Implen Nano Photometer NP60 spectrophotometer (Implen, Munich, Germany). Fifteen individual plants of each mutant line were used to estimate the genetic heterogeneity.

### Genome-specific PCR

Allelic forms of the *B*. *napus* FAD3 genes were identified by PCR amplifications of the gene fragments comprising wild-type and mutation sites followed the detection of the mutant alleles by the microsequencing method (SNaPshot) with locus-specific primers.

Target DNA fragments were amplified in two independent reactions with genome-specific primer pairs FAD3Af/FAD3Ar for the BnaA.FAD3 gene and FAD3Cf/FAD3Cr for the BnaC.FAD3 gene as it was described earlier [49]. Amplification was carried out on SimpliAmp Thermal Cycler (Applied Biosystems, Foster City, USA) in following conditions: 4 min at 95°C followed by 30 steps with 30 s at 95°C, 30 s at 55°C and 30 s at 72°C, and with the final elongation step for 30 min at 72°C. Reaction mix included 100 ng of genomic DNA template, 1 μM dNTP, 1.5 mM MgCl2, 10x PCR-buffer (650 mM Tris-HCl, 166 mM (NH4)2SO4, 0,2% tween 20, pH 8.8), 0.25 μM each primer, and 1 U Taq polymerase (Primetech, Minsk, Belarus) in a total volume of 25 μl. PCR products were separated by electrophoresis in 1.5% agarose gel with an addition of ethidium bromide solution to a final concentration of 0.5 μg/ml at a voltage of 100 V with the use of 100 bp Plus DNA-ladder (Thermo Scientific, Vilnius, Lithuania). After amplification, post-PCR purification was performed as follows: 5 μl of the PCR product was incubated with 1 U of FAST alkaline phosphatase and 2 U of exoI (Thermo Fisher Scientific, Vilnius, Lithuania) for 1 h at 37°C, followed by 15 min at 80°C for enzyme inactivation.

### SNaPshot analysis

The amplified on the first step fragments were used for the detection of FAD3 mutant and wild-type alleles by SNaPshot technique. In the SNaPshot analysis, previously described primers mutA-1f and mutC-45F [49] modified with a poly-A tail, were used. To discriminate these fragments, the s550 high density size standard for fragment analysis (Synthol, Moscow, RF) was used. Primer extension reactions were carried out independently for FAD3A and FAD3C in a final volume of 10 μl containing 2 μl exoI/FAST treated PCR product (5–50 ng DNA) as a template, 2 μl of the SNaPshot Ready Reaction Mix (Applied Biosystems, Foster City, USA) and 0.2 μM primer. The following amplification protocol was applied: 35 cycles of 10 s at 95°C, 5 s at 50°C and 30 s at 60°C. After the extension reaction, PCR products were treated with FAST alkaline phosphatase (1 unit per sample) for 1 h at 37°C. For electrophoresis, 0.5 μl of the purified primer extension reaction products were combined and mixed with 9 μl of Hi-Di (highly deionized) formamide and 0.5 μl of s550 size standard (Synthol, Moscow, Russia), denatured for 5 min at 95°C and separated by capillary electrophoresis on an ABI Prism 310 Genetic Analyser (Applied Biosystems, Foster City, USA) using POP6 polymer. Alleles of the FAD3A and FAD3C were scored using Gene Mapper 4.1 software (Applied Biosystems, Foster City, USA). The presence of polymorphic alleles was visualized by colour depending on the included in the SNaPshot-PCR product ddNTP which carried the corresponding fluorescent label:

- A–dR6G label–green
- C–dTAMRA label–black
- G–dR 110 label–blue
- T(U)–dROX label–red

Considering that the alleles of FAD3 genes differed from each other by one nucleotide (FAD3A –C; fad3A –T; FAD3C –G; fad3C –A), the following coloured peaks were visualized as a result of SnaPshot-PCR with a single locus-specific forward primer:

- FAD3A (wild-type allele)–black
- fad3A (mutant allele)–red
- FAD3C (wild-type allele)–blue
- fad3C (mutant allele)–green

### Chromosome spreads

For chromosome spread preparation, rapeseed root tips (1–0.5 cm) were incubated (16–24 h) in ice-cold water with 1 μg/mL of 9-AMA (Sigma, St. Louis, USA) to inhibit chromosome condensation process and accumulate prometaphase chromosomes [50]. Then, the roots were treated in ethanol: glacial acetic acid fixative (3:1) for 48 h at room temperature and after that stored at −20°C until use. Chromosome spreads were prepared according to the technique described previously [51].

For meiotic chromosome preparation, young floral buds (prefoliation) were fixed in ethanol:acetic acid (3:1) fixative for 30 min at 4°C and then chromosome spreads were prepared as previously described [51]. After freezing in liquid nitrogen, the cover glasses were removed, and the slides were stored in 96% ethanol at −20°C until use.

### DNA probe preparation and FISH

Following probes were used for FISH:

1. pTa71 containing a 9 kb long repeated DNA sequence of common wheat including 18S- 5.8S-26S rDNA [52];
2. pTa794 containing a 420 bp long repeated DNA sequence of wheat including 5S rDNA [53].

DNA probes were labelled directly with SpectrumRed or SpectrumAqua fluorochromes (Abbott Molecular, Wiesbaden, Germany) by nick translation according to manufacturer’s protocol. FISH procedure was performed as described previously [54]. After hybridization (16–20 h), the slides were washed twice with 0.1xSSC at 44 C for 10 min, twice with 2xSSC at 44 C for 5 min followed by a 5-min wash in 2xSSC and three washes in PBS for 3 min each at room temperature. Then the slides were dehydrated through a graded ethanol series and air dried.

### DAPI-banding

After the FISH procedure, chromosome slides were stained with 0.1 μg/mL DAPI (4′,6-diamidino-2-phenylindole) (Serva, Heidelberg, Germany) in Vectashield mounting medium (Vector laboratories, Peterborough, UK). DAPI-banding analysis was used as an additional parameter for the identification of individual chromosomes [46, 47].

### Chromosome analysis

The slides were examined using Olympus BX61 epifluorescence microscope (Olympus, Tokyo, Japan) combined with a monochrome CCD camera (Cool Snap, Roper Scientific Inc., Tucson, USA). The captured images were processed with Adobe Photoshop 10.0 software (Adobe Systems Inc., Birmingham, USA). At least 30 plants of each line and 15 metaphase plates of each plant were analyzed. In karyotypes, the cytological numerical designation of the chromosomes of A and C subgenomes was according to Levan’s criterion [55]. Additionally, the identification of chromosomes and genome affiliation were performed based on the chromosome morphology, revealed chromosome markers as well as earlier described data [46, 47, 56, 57]. The meiotic chromosome preparations were analyzed as described previously [51].

### Analysis of pollen

The examination of pollen grains was performed with the use of a scanning electron microscope (SEM) JEOL JSM– 6380LA (accelerating voltage 20 kV, SEI mode) (Jeol, Tokyo, Japan). In each line, pollen grains were collected from six plants (three flowers from the main inflorescence). Fresh pollen was mounted on carbon adhesive tape. The analysis of pollen grains was performed with the use of SEM Control User Interface, Version 7.11 (Jeol, Tokyo, Japan). For each line, ten ocular views (250 x) of pollen grains were analysed. Statistical data analysis was performed using standard functions of Microsoft Excel 2013.

## Results

### Morphological characterization

Within the M2-M3 progeny, a segregation of morphological traits was found, and plants of *B*. *napus*-like and *B*. *rapa*-like morphotypes displaying distinct morphological differences were revealed. In M4-M5 generations, the progeny of *B*. *napus*-type plants presented constant morphotypes. Within the progeny of the *B*. *rapa*-type line, further segregation of morphological traits was observed, and both *B*. *rapa*- and *B*. *napus*- (up to 12%) morphotypes were detected.

**Fig 1:**
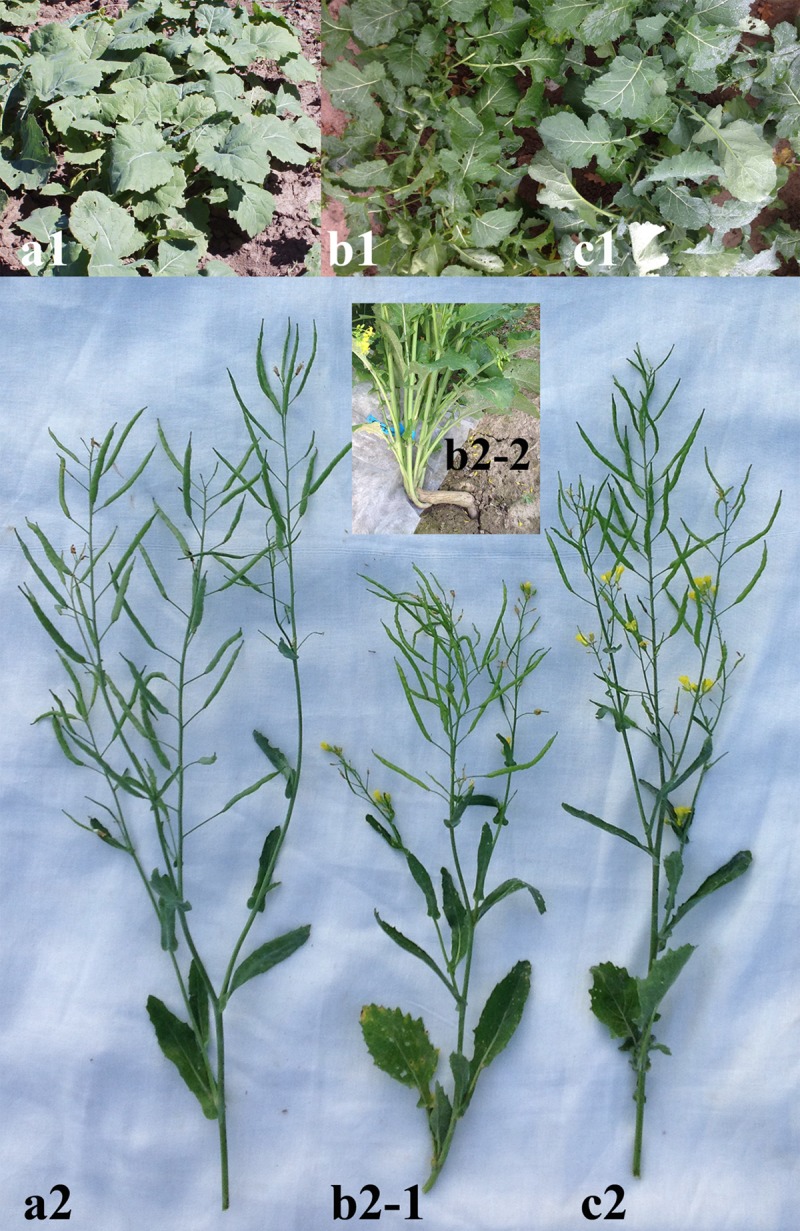
Plants of original *B*. *napus* cv. Vikros and its mutant progeny. Plants of *B*. *napus* cv. Vikros (a1), mutant plants of *B*. *rapa*-type (b1) and *B*. *napus*-type (c1) at the rosette vegetative growth stage; position and shape of siliqua in *B*. *napus* cv. Vikros (a2), in plants of *B*. *rapa*-type (b2-1) and *B*. *napus*-type (c2); a plant of *B*. *rapa*-type with a long hypocotyl (b2-2).

At the stage of the third pair of true leaves, these morphological differences became more evident. Plants of the *B*. *rapa*-like morphotype had tender, thin, round and puberulent leaf blades and bright green (non-glaucous) leaves, stems and siliqua (Fig 1). The pubescence disappeared at the stage of the fifth pair of true leaves. Rapeseed-like plants had more coriaceous and smooth leaf blades and glaucous leaves, stems and siliqua (Fig 1).

In most mutant plants, we observed moderate decrease in the mean value of plant height compared to the original cultivar. However, this character was highly variable (Table 1). The plants of *B*. *rapa*-type had longer hypocotyl (Fig 1) and were more liable to lodging at the stage of early flower bud formation if compared with the original cultivar and rapeseed-type plants. Then, the leading shoot checked in growth, and the plant height in such plants became contingent on first-order shoot development. Basal first-order shoots in *B*. *rapa*-type plants, were well-developed and grew subopposite from the hypocotyl (vs. in *B*. *napus*-type plants, they were also well-developed but grew from the root neck). In plants of *B*. *rapa*-type, shoots III were also observed though side shoots II were less developed compared to *B*. *napus*-type (Table 1). Siliqua in *B*. *rapa*-like plants were thinner and grew more vertical (vs. in *B*. *napus*-type plants, the angle was about 45°) (Fig 1).

**Table 1:** Vegetative parameters in *B*. *napus* cv. Vikros and M5 plants.

Traits	*B*. *napus* cv. Vikros	M5 generation		
		Constant	Segregated	
		*B*. *napus*-type	*B*. *rapa*-type	*B*. *napus*-type
Plant height (cm)	88.9±7.5	87.2±6.2	81.4±7.6	84.3±6.6
Number of shoots I	4.4±1.2	5.4±1.1	6.1±1.8	6.4±1.2
Number of shoots II	4.3±1.3	4.1±1.6	5.4±1.7	7.9±1.6*
Fertile pollen grains (%)	80±3	69±5*	46±7*	68±5*
Number of seeds per silique	25.0±3.8	25.2±3.1	21.1±4.7	25.7±2.9
Seed yield per plant (g)	27.6±3.3	26.3±3.1	15.7±3.5*	21.7±6.9
Weight of 1000 seeds (g)	3.5±0.4	3.4±0.3	2.6±0.5*	3.4±0.5

Each meaning represents the mean value ± standard deviation

*The values are significantly different at P ≤ 0.05

**Fig 2:**
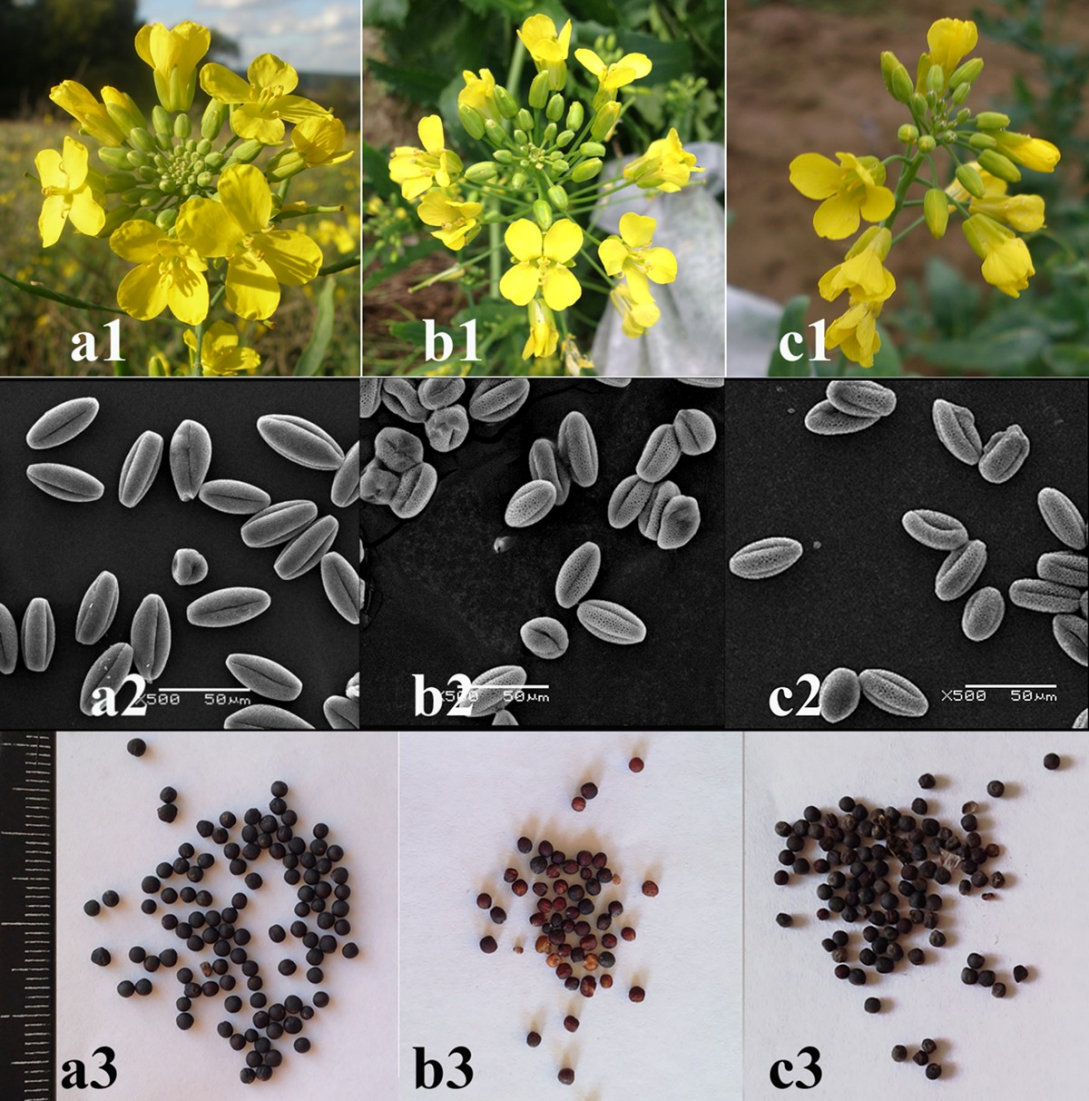
Vegetative parameters in *B*. *napus* cv. Vikros and mutant plants. Inflorescences of *B*. *napus* cv. Vikros (a1), plants of *B*. *rapa*-type (b1) and *B*. *napus*-type (c1); SEM images of pollen grains in *B*. *napus* cv. Vikros (a2), in plants of *B*. *rapa*-type (b2) and *B*. *napus*-type (c2); seeds of *B*. *napus* cv. Vikros (a3), plants of *B*. *rapa*-type (b3) and *B*. *napus*-type (c3). Scale bar– 50 μm.

All studied plants had yellow racemose inflorescences. In mutant plants, inflorescences were shorter and few-flowered. In plants of *B*. *rapa*-type, flowers were a little smaller and the flower colour was lighter than in rapeseed-like plants (Fig 2). In all studied plants, the pollen grains were tricolpate (typical for *Brassicaceae*) (Fig 2). However, more imperfect and/or deformed pollen grains were revealed in plants of *B*. *rapa*-type compared to the original cultivar and rapeseed-like plants (Fig 2, Table 1). Also, in plants of *B*. *rapa*-type, the number of seeds per silique was more variable; seeds were red-brown, irregular-shaped and smaller in size; and seed productivity was less if compared with the plants of rapeseed-type (Fig 2, Table 1).

### Biochemical profile and fatty acid composition

**Fig 3:**
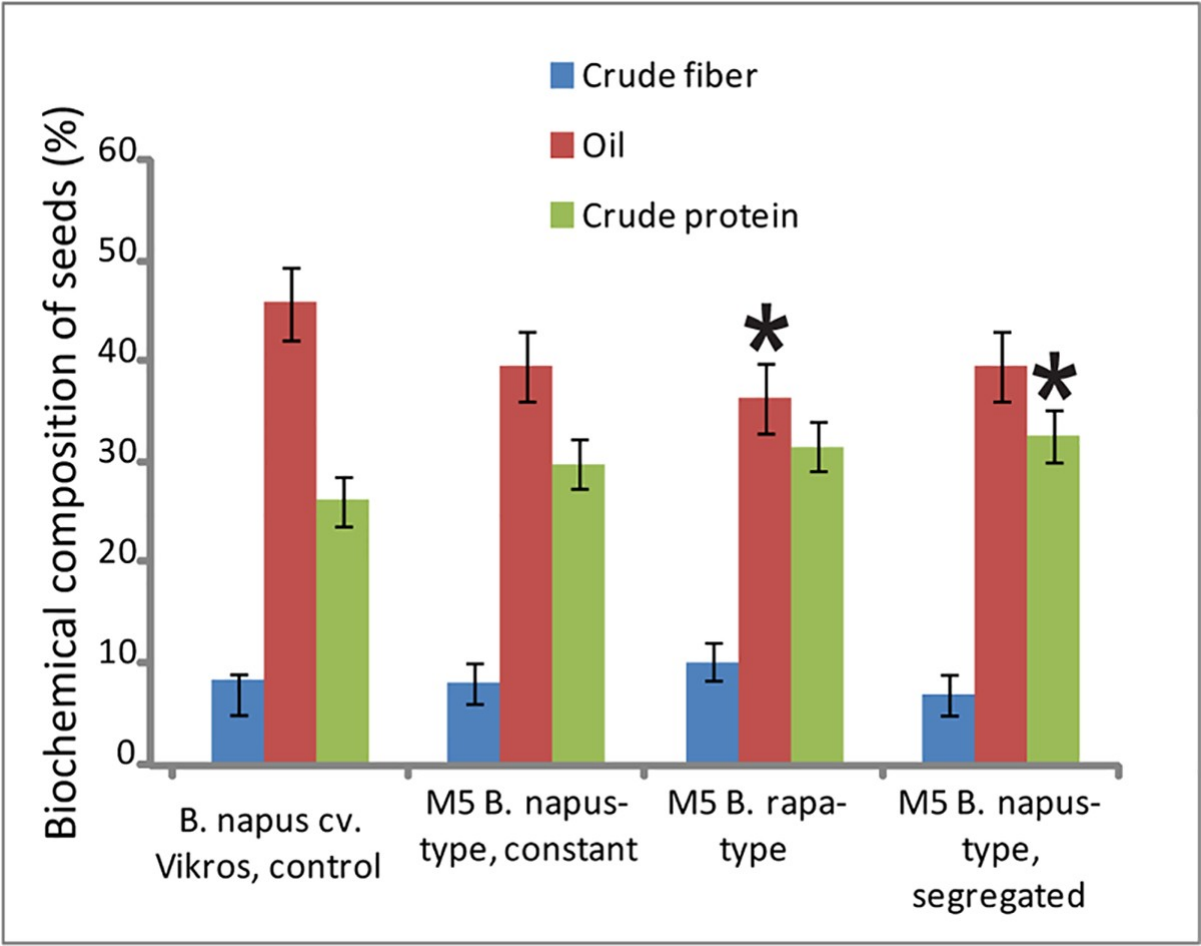
Biochemical composition of seeds in *B*. *napus* cv. Vikros and M5 plants. Contents of crude fiber (blue), oil (red) and crude protein (green) (the vertical axis, %) in the original cultivar and EMS populations (the horizontal axis).

Biochemical analysis of seeds showed that the oil content in the mutant lines was rather low (39–42%) especially, in *B*. *rapa*-type plants (28–39%), and the protein content was high (about 30%, in plants *B*. *rapa-* and segregated *B*. *napus-*type it reached 35%). Biochemical analysis also revealed a high level of variability in seed crude fiber contents among the mutant plants of different morphotypes (Fig 3).

The analysis of fatty acid (FA) compositions in seeds showed that in mutant plants, the palmitic (C_16:0_) fatty acid was synthesized more extensively compared to the original *B*. *napus* cv. Vikros. The contents of the other main fatty acids were roughly comparable with the original cultivar. Besides, small amount (≤ 2%) of 7–8 other fatty acids which were not typical for rapeseed (С_8:0_, С_14:0_, С_16:2_, С_16:3_, С_17:0_, С_17:1_, С_24:0_, С_24:1_) were detected in the studied rapeseed plants (Table 2).

**Table 2:** Fatty acid composition of seeds in *B*. *napus* cv. Vikros and M5 plants.

Fatty acid (%)	*B*. *napus* cv. Vikros	M5 generation		
		Constant	Segregated	
		*B*. *napus*-type	*B*. *rapa*-type	*B*. *napus*-type
C_16:0_	2.73±0.32	5.24±0.53*	3.90±0.22*	4.01±0.37*
C_16:1_	0.15±0.03	0.25±0.03	0.13±0.02	0.14±0.01
C_18:1_	63.87±1.54	59.41±2.36	60.96±0.97	59.03±1.08
C_18:2_	19.66±0.25	23.41±1.57	22.49±2.48	21.32±0.72
C_18:3_	9.01±0.90	7.16±0.23	8.51±0.28	8.42±0.64
C_20:0_	0.5±0.06	0.84±0.06	0.56±0.07	0.62±0.03
C_20:1_	1.46±0.17	1.28±0.12	1.31±0.04	1.28±0.05
C_22:0_	0.30±0.17	0.44±0.04	0.32±0.03	0.30±0.02
Other fatty acids	2.26±0.08	1.99±0.05	1.20±0.03	1.86±0.01

Each meaning represents the mean value ± standard deviation

*The values are significantly different at P ≤ 0.05

### SNaPshot analysis

The SNaPshot analysis was performed for 20 M4-M5 plants of different types including plants with abnormal karyotypes and also the original cultivar. This analysis detected only wild-type alleles of FAD3 genes in both A and C subgenomes in all studied samples (Table 3).

**Table 3:** The results of SNaPshot detection of mutant and wild-type alleles of FAD3 genes in *B*. *napus* cv. Vikros and M4-M5 plants.

Number	Sample	Fragment		Peak		Allele	
	description	length		colour		type	
		A-genome	C-genome	A-genome	C-genome	A-genome	C-genome
1	*B*. *napus* cv. Vikros	63.14	65.1	Black	Blue	WT	WT
2	M4-12 (seedlings)	63.15	65.12	Black	Blue	WT	WT
3	М4–2 (seedlings)	63.14	65.1	Black	Blue	WT	WT
4	М4–23 (seedlings)	63.2	65.15	Black	Blue	WT	WT
5	М4–9 (seedlings)	63.05	65.1	Black	Blue	WT	WT
6	М4–4 (seedlings)	63.14	65.1	Black	Blue	WT	WT
7	М4–4 (*B*. *rapa-*type)	63.19	65.18	Black	Blue	WT	WT
8	M5-9 (*B*. *napus-*type)	63.14	65.1	Black	Blue	WT	WT
9	M5-9 (*B*. *rapa*-type)	63.14	65.1	Black	Blue	WT	WT
10	M5-11 (*B*. *napus-*type)	63.19	65.18	Black	Blue	WT	WT
11	M5-11 (*B*. *rapa-*type)	63.14	65.1	Black	Blue	WT	WT
12	M5-17 (*B*. *napus-*type)	63.19	65.09	Black	Blue	WT	WT
13	M5-17 (*B*. *rapa*-type)	63.14	65.1	Black	Blue	WT	WT
14	M5–7 (*B*. *napus-*type)	63.09	65.07	Black	Blue	WT	WT
15	M5-7 (*B*. *rapa-*type)	63.19	65.18	Black	Blue	WT	WT
16	M5-12 (*B*. *napus-*type)	63.19	65.1	Black	Blue	WT	WT
17	M5-12 (*B*. *rapa-*type)	63.14	65.01	Black	Blue	WT	WT
18	M5-3 (*B*. *napus-*type)	63.15	65.09	Black	Blue	WT	WT
19	M5-3 (*B*. *rapa-*type)	63.14	65.1	Black	Blue	WT	WT
20	M5-2 (*B*. *rapa-*type)	63.11	65.06	Black	Blue	WT	WT
21	M5-2 (*B*. *napus-*type)	63.14	65.1	Black	Blue	WT	WT

### Chromosomal structural variations in the EMS populations

**Fig 4:**
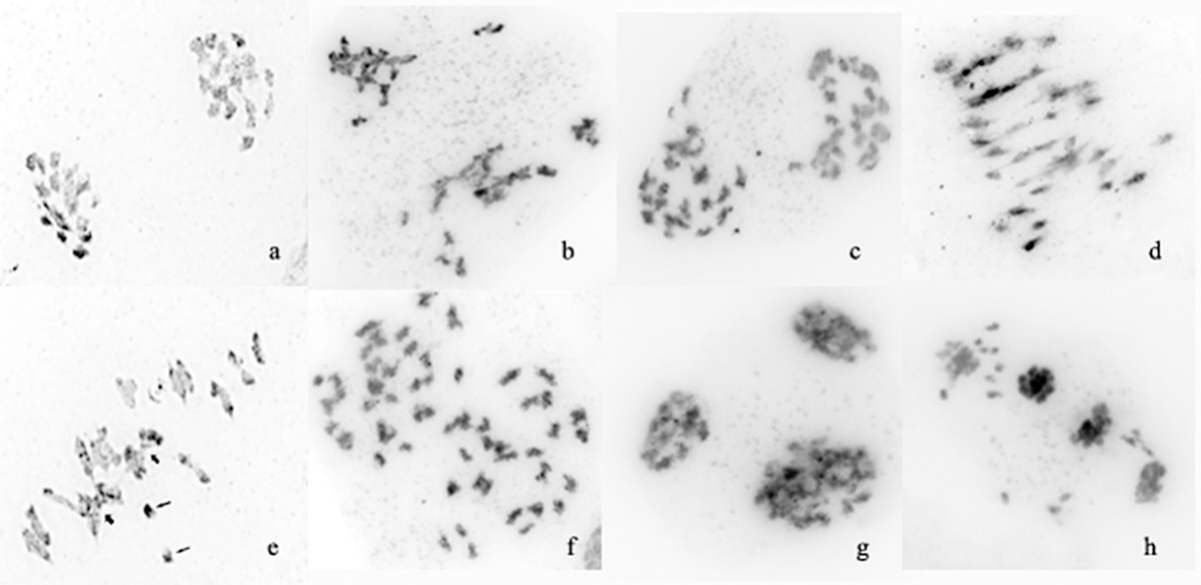
Meiosis in maternal pollen cells in *B*. *napus* cv. Vikros and mutant plants. (a) A-I, 19^II^; (b) several chromosomes are localized outside the metaphase plate; (c) A-I, chromosome lagging; (d) A-I, chromosomal bridges; (e) M-I, 14^II^+2^IV^(short arrows)+2^I^ (long arrows); (f) A-I, chaotic disjunction and chromosome lagging; (g) three-polar configuration with chromatin agglutination; (h) asynchronous division within one meiocyte.

In most studied maternal pollen cells of *B*. *napus* cv. Vikros, regular meiotic chromosome behavior with normal chromosome disjunction and nineteen bivalents (19^II^) was observed (Fig 4A). Besides, in the reduction division, few common meiotic abnormalities were detected. As an example, the occurrence of some chromosomes outside the metaphase spread is shown in Fig 4B. However, the cumulative percentage of these irregularities in maternal pollen cells was nonessential (~1.5%).

In both constant and segregated populations of *B*. *napus*-type plants, common meiotic abnormalities were detected in 0.15–5.1% of the maternal pollen cells. For instance, chromosome lagging and chromosome bridges at anaphase I are shown in Fig 4C and Fig 4D, correspondingly. Besides, at anaphase II, the spindle function related abnormalities (asynchronous division and lagging) were also detected in maternal pollen cells.

In the *B*. *rapa*-type plants, the cumulative percentage of common meiotic abnormalities in maternal pollen cells was ranged from 0.15% to 11.8%. For example, univalents and quadrivalents in the reduction devision (M-1) as well as chaotic disjunction and chromosome lagging at A-I are presented in Fig 4E and Fig 4F, correspondingly.

In one M5 plant of *B*. *rapa*-type, multiple meiotic abnormalities (elimination of chromosome groups at anaphase-telophase I, micronuclei in dyads, chromosome elongation and chaotic chromosome distribution at metaphase II, chromatin agglutination, three-polar configurations and asynchronous division within one meiocyte) were revealed in 0.17%– 35.7% of the studied maternal pollen cells. For example, a three-polar configuration with chromatin agglutination and asynchronous division within one meiocyte are shown in Fig 4 g and Fig 4H, correspondingly.

**Fig 5:**
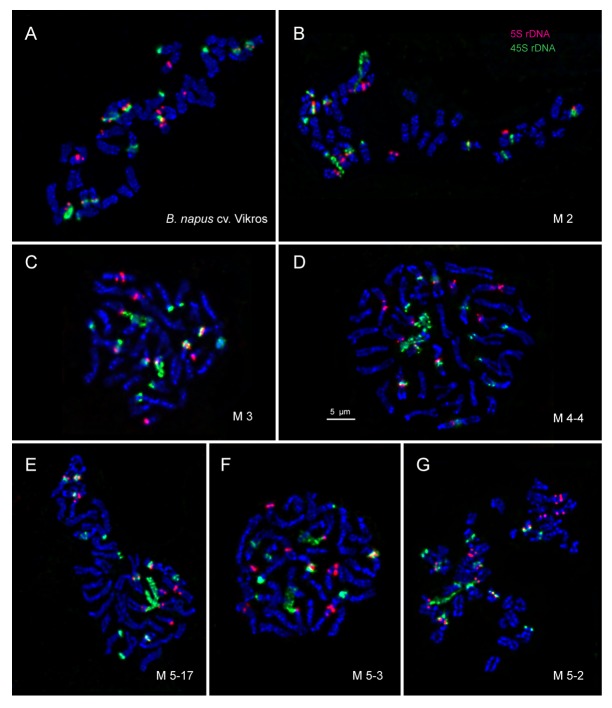
FISH-based localization of 45S and 5S rDNA on chromosomes of *B*. *napus* cv. Vikros and M2-M5 mutant plants. Metaphase plates of *B*. *napus* cv. Vikros (A), M2 plant of *B*. *napus*-type (B), M3 plant of *B*. *rapa-*type (C), M4-4 plant of *B*. *rapa*-type (D), M-17 plant of *B*. *napus-*type (segregated) (E), M5-3 plant of *B*. *rapa-*type (F), M5-2 plant of *B*. *rapa-*type (G). The correspondent probes and their pseudo-colours are specified in the upper right-hand corner. DAPI-banding (blue). Scale bar– 5 μm.

**Fig 6:**
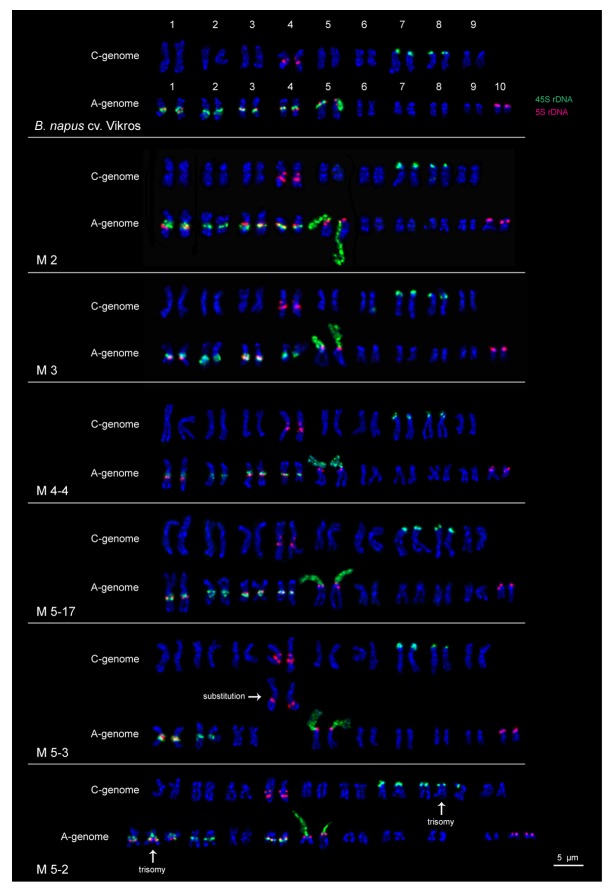
Karyotypes of *B*. *napus* cv. Vikros and M2-M5 mutant plants. Karyograms of the metaphase plates shown in Fig 5 after DAPI-banding (blue) and FISH with 45S (green) and 5S rDNA (red). Scale bar– 5 μm.

In the original cultivar and most studied M2-M5 plants, rapeseed karyotypes with 2n = 38 chromosomes were observed. The exception was one M5 plant with 2n = 40 chromosomes (Figs 5 and 6).

In karyotypes of the original cultivar, FISH analysis revealed separate 45S rDNA sites in the secondary constriction regions (subtelomere positions of the short arms) of two large chromosome pairs 7 and 8 (C subgenome) and also in the pericentromeric region of one middle-sized chromosome pair 2 (A subgenome). Separate 5S rDNA sites were detected in the pericentromeric and interstitial positions (the long arm) of one large chromosome pair 4 (C subgenome) and in the subtelomere region of the short arm of the smallest chromosome pair 10 (A subgenome). Co-localized 45S and 5S rDNA sites were found in the pericentromeric region of middle-sized chromosome pairs 1, 3 and 4 (A subgenome) and also in the secondary constriction region (subtelomere positions of the short arms) of the pair of a middle-sized chromosome pair 5 (A subgenome) (Figs 5 and 6).

In karyotypes of most studied mutant plants, patterns chromosomal distribution of 45S and 5S rDNA were similar to those observed in the original cultivar with the exception of one M3 plant of *B*. *rapa*-type having only separate 45S rDNA sites on chromosome pairs 4 (A subgenome); one M5 plant of *B*. *rapa*-type with double trisomy (2n = 40) and also one M5 plant of *B*. *rapa*-type with a homeologous substitution of one chromosome pair (4) between A and C subgenomes (Figs 5 and 6).

## Discussion

EMS is included among the so-called 'supermutagens' which can be used to generate the important recessive and dominant genomic mutations at a high rate and thereby create a basis for useful genetic variations required for plant breeding programs [23, 58–59]. EMS mutagenesis is an effective approach to create mutations in genes of the polyploid species such as *B*. *napus*. These mutagens was found to induce non-lethal point DNA mutations which could be retained in the genome due to its capacity for self-pollination [60]. These induced genetic variations correlate to variability in agronomic and phenotypic traits in rapeseed mutant populations [61–63]. In the present study, EMS mutagenesis induced extensive morphological diversity among mutant progeny of canola *B*. *napus* cv. Vikros. As a result, we could successfully select EMS populations of *B*. *napus*- and *B*. *rapa-*morphotypes displaying distinct differences in morphological and agronomic traits. Within the progeny of the *B*. *rapa*-type line, further segregation of morphological traits was observed indicating that EMS had induced the heterozygous mutations in genomes of *B*. *rapa*-type plants, and both *B*. *rapa*- and *B*. *napus*- (up to 12%) morphotypes were revealed. As it was quite possible that the mutagenesis could result in genotypic differences between constant and segregated populations of *B*. *napus*-type, we performed comparative analysis among the EMS populations of different types. Currently, producing short-stem lines is a high-priority task, and due to breeding efforts for growth limitation of lateral meristems in joints of a plant stem, plant height is considered to be an important agronomic trait. The decrease in plant height was described earlier in several EMS mutagenized populations of crops including *Brassica* species [63]. In the studied EMS populations of different types, we observed moderate decrease in plant height and also high variability of this feature. These findings indicate that plant height could probably be reduced by a further selection process in the EMS progeny.

Also, plants of *B*. *rapa*-type and segregated *B*. *napus*-type plants were found to have more extensively developed shoots I and shoots II. Probably, due to high density of plant tillers, poor flowering, lower number of siliqua and lower level of seed productivity were observed in those plants compared to plants of the original cultivar and constant rapeseed-type.

Biochemical analysis showed that seeds of the studied mutant plants were high-protein and low in oil which makes them useful for feed production. Also, seeds of *B*. *rapa-*type plants had the highest crude fiber content, but this character was more variable compared to the protein contents in the seeds.

Different fatty acid components of rapeseed oil make it best suited to particular uses. Canola ‘00’ (low erucic acid and low glucosinolate) produces seeds that are used to generate excellent edible oil that is lower in saturated fat and higher in omega-3 fatty acids than most other commercially available oils [64]. These attributes have been shown to have a significant positive impact on human health, reducing diseases such as cancer, heart disease and some neurological disorders [65, 66]. EMS mutagenesis can induce genetic changes in plants and modify the levels of fatty acids in seed oil [60]. In this study, however, the treatment of canola seeds with EMS at low concentration did not influence the contents of main fatty acids in canola seeds with the exception of a palmitic (C_16:0_) acid which level was higher compared to the original cultivar. One of the rapeseed breeding goals is to obtain genotypes producing naturally stable oil. Particularly, a low content (≤10%) of the linolenic acid prevents oxidation and rancidification of seed oil which is important for healthy food production [60, 67]. Besides, high stability of the oil with low linolenic acid content makes it an important source of raw material for biofuel production. Genetic analyses revealed that the fatty acid composition of rapeseed varied depending on the allelic composition of FAD3 genes as well as the ratio of mutant fad3a, fad3c alleles and FAD3, FAD3A, FAD3C wild-type alleles [49, 67]. Moreover, single-nucleotide mutations detected in mutant rapeseed lines resulted in a decrease in the content of linolenic acid in rapeseed oil [39, 49]. SNaPshot analysis using SNP markers is an effective approach for detecting mutant alleles of the FAD3 genes in *B*. *napus* [49, 67]. In the present study, the performed SNaPshot analysis did not detect any single-nucleotide polymorphisms in FAD3 genes in both A and C subgenomes indicating the homozygous state of these genes in the studied lines. Considering also that the original canola cultivar and the plants of *B*. *rapa*- and *B*. *napus-*morphotypes had related meanings of linolenic (C_18:3_) fatty acid contents (8–10%), our results showed that mutagenesis did not influence the stability of this essential fatty acid in the obtained mutant lines.

Chemical mutagens can influence the plant genome and cause the meiotic disorders manifested themselves as typical anaphase aberrations (chromosome fragments, bridges, lagging, etc.) as well as fragmentation, nondisjunction, chromosome stickiness and other abnormalities [17, 68]. In most studied here maternal pollen cells of the original rapeseed cultivar and mutant lines, normal chromosome disjunction (19:19) was observed. However, typical meiotic abnormalities including chromosome fragments, chaotic chromosome disjunction and lagging at anaphase I; occurrence of some chromosomes outside the metaphase spread and bridges were also revealed. Chromosome nondisjunction, occurred at anaphase I, is considered to be a serious meiotic abnormality which resulted in chromosome loss as well as unequal distribution of genetic material. These disorders could appear due to the paracentric inversions as previously described in tomatoes and *Nigella sativa* [69, 70].

Besides, deviations from the normal bivalent conjugation could be displayed as univalent and multivalent formation at metaphase I stage [71]. In this study, univalents at diakinesis were also detected in maternal pollen cells of the studied mutant plants. The mutagen-induced univalent formation was supposed to be a result of chromosome structure changes followed by the reduction of chiasma frequency due to restriction of pairing to homologs [72].

In the original *B*. *napus* plants, the cumulative percentage of meiotic irregularities in maternal pollen cells was nonessential (~1.5%). However, the percentage of cells with meiotic disorders was higher in the studied plants rapeseed-type (up to 5.1%) and *B*. *rapa*-type (up to 11.8%) compared to the original cultivar. In one M5 plant of *B*. *rapa*-type, multiple meiotic abnormalities including elimination of chromosome groups at anaphase-telophase I, micronuclei in dyads, chromosome elongation and chaotic chromosome distribution at metaphase II, chromatin agglutination, three-polar configurations and asynchronous division within one meiocyte were revealed. The analysis of meiotic chromosome behaviour indicated that in plants of the obtained EMS populations, various chromosome rearrangements could occur. Probably, the observed high level of phenotypic variability could also be related to these chromosomal variations. However, zygotes with chromosome abnormalities (appeared due to disorders during meiosis) were shown not always to produce viable seeds, and most meiotic abnormalities were eliminated before the tetrade stage and therefore, do not influence the pollen quality [73]. However, several meiotic irregularities, such as chromatin agglutination, as well as high level of abberrations (35.7%) revealed in this sample could reduce the quality and fertility of pollen and subsequently, result in reductions in seed yield.

The amphidiploid genome of *B*. *napus* consists of closely related A and C subgenomes [44, 74–76] which display numerous deviations from parental *Brassica* species additivity [77–79]. Consequently, *B*. *napus* is considered to be an important model species to study the processes of genomic reorganizations in recently formed polyploids [15, 80–81]. The examples of such processes could be different chromosomal rearrangements and intragenomic substitutions observed in natural and resynthesized rapeseed lines which could probably be related to the maintenance of genomic stability [15, 45]. Also, it was previously shown that an enhanced genome instability in resynthesized rapeseed lines developed under the pressure of selection resulted in chromosome rearrangements or/and deletions and even elimination of the whole parental genome in hybrids in the succeeding generations [47]. Besides, intraspecific polymorphism in pattern of chromosomal distribution of 45S and 5S rDNA was previously described for *B*. *napus* [46]. In this study, the molecular cytogenetic analysis of the original *B*. *napus* cultivar and obtained mutant lines of *B*. *rapa*- and *B*. *napus*-morphotypes indicated a high degree of karyotypic stability despite the fact that the cumulative percentage of microsporocytes with meiotic disorders was higher in mutant plants compared to the original cultivar. FISH analysis showed that all the studied karyotypes in *B*. *napus*-type plants and most karyotypes in *B*. *rapa*-type plants did not differ in chromosome number, morphology and pattern of 45S and 5S rDNA chromosomal distribution from the original cultivar. However, among M3-M5 progeny of *B*. *rapa*-type, chromosomal reorganizations including variations in number of 45S and 5S rDNA, trisomy and substitutions between homeological chromosomes were also revealed. It should be noted that the observed chromosomal reorganizations correlated to the higher levels of different meiotic abnormalities, differences in plant morphology and also low seed productivity detected in *B*. *rapa*-type progeny, and this could be related to the EMS induced mutations. Different cytogenetical abnormalities induced by EMS mutagenesis were observed earlier in tomatoes and *Nigella sativa* [71, 72]. Our findings demonstrate that rapeseed breeding via chemical mutagenesis could result in cytogenomic instability in the obtained mutant progeny, and therefore, should include karyotype examination.

Thus, molecular cytogenetic analysis of the original *B*. *napus* cv. Vikros and its EMS mutagenized progeny indicated that the processes of mutagenesis and also selection for morphological and agronomic traits did not induce changes in chromosomal structure of both constant and segregated mutant lines of *B*. *napus-*type, and these mutant lines could be a basis for further rapeseed improvement. The revealed structural chromosomal reorganizations in karyotypes of the mutant plants of *B*. *rapa-*type showed that it can be useful for the development of rapeseed forms with trisomy and also chromosome addition/substitution lines. Such aneuploidy lines are important for rapeseed breeding as they provide the opportunity to produce introgression lines and also offer the way to check heterologous gene expression and interaction between recipient genome and donor chromosomes in plants [82–84].

## Conclusions

In the present study, EMS mutagenesis induced extensive diversity in morphological and agronomic traits among mutant progeny of canola *B*. *napus* cv. Vikros resulted in selection of EMS populations of *B*. *napus*- and *B*. *rapa-*morphotypes. The obtained unique data on phenotypic, biochemical and cytogenomic variability within these populations showed distinct differences among them. The mutant plants with abnormal karyotypes revealed within the EMS populations indicate that rapeseed breeding by chemical mutagenesis can induce chromosome instability in the mutant progeny, and therefore, it should include karyotype examination. Our findings demonstrate that EMS at low concentrations has great potential in rapeseed improvement.
